# Travel‐related gastrointestinal diseases: Assessment and management

**DOI:** 10.1002/puh2.30

**Published:** 2022-11-02

**Authors:** Dominic Butler, Ramona McLoughlin, Gerard T. Flaherty

**Affiliations:** ^1^ School of Medicine College of Medicine Nursing and Health Sciences University of Galway Galway Ireland; ^2^ Department of Gastroenterology University Hospital Galway Galway Ireland; ^3^ School of Medicine International Medical University Kuala Lumpur Malaysia

**Keywords:** travel health, travellers’ diarrhoea, persistent abdominal symptoms

## Abstract

**Introduction:**

Gastrointestinal illnesses are among the most common health problems affecting travellers returning from international travel. Its public health implications include economic healthcare costs and the risk of promoting antimicrobial resistance. This narrative review aims to critically appraise the literature concerning gastrointestinal diseases in travellers and outlines a practical approach to aid clinicians in their assessment and management.

**Methods:**

The Medline (PubMed) and SCOPUS databases were searched for articles relating to gastrointestinal issues in returning travellers. Articles were primarily restricted to those published in the last 5 years. Only studies on human subjects were included.

**Results:**

Diarrhoea is the most common gastrointestinal symptom experienced by approximately 40%–60% of travellers, but abdominal pain, bloating and fatigue are also common. Diarrhoeagenic *Escherichia coli* species remain the most common cause of travellers’ diarrhoea (TD), with parasitic diseases predominating in those with persistent symptoms. Molecular diagnostic techniques enable highly sensitive simultaneous testing for a multitude of enteropathogens, though more research is required to confirm the pathogenic significance of organisms identified. Further research into poorly absorbable antibiotics and novel therapies such as phage treatment may help to alleviate the burden of antimicrobial resistance. There is a role for empirical anti‐parasitic therapy in selected cases.

**Conclusion:**

Clinicians across a range of medical specialities should be familiar with the presentation, aetiology and management of gastrointestinal illness in the returning traveller. Efforts to improve destination sanitation and hygiene infrastructure may serve to reduce the burden of preventable illness in this setting.

## INTRODUCTION

Gastrointestinal diseases are among the most common health problems affecting returned travellers, with one study reporting that gastrointestinal disease accounted for 37% of presentations in a sample of over 10, 000 unwell returning travellers [1]. The public health implications of this health problem are linked to the frequency of the condition, the associated health economic costs, and the potential for importation and spread of multi‐drug resistant organisms from areas of higher prevalence. This narrative review aims to summarise and critically appraise the literature concerning gastrointestinal diseases in returning travellers. While topics such as traveller's diarrhoea and the microbiome in travellers are well studied, we summarise the overall context of gastrointestinal disease and outline a practical approach to aid clinicians who encounter returning travellers with gastrointestinal issues. It may also serve as a useful resource for personnel working in hospitality and tourism settings in low‐ or middle‐income countries.

## SEARCH STRATEGY

Medline (PubMed) and SCOPUS databases were searched for articles relating to gastrointestinal issues in returning travellers, using various combinations of relevant search terms, including “travel*” and (“abdom*” or “gastrointestin*” or “diarrh*” or “dysenter*” or “naus*” or “vomit*” or “microbio*”). Articles were primarily restricted to those published in the last 5 years from January 2017 through July 2022 and written in English. Reference lists of the articles were screened for studies not discovered in the primary search.

## TRAVELLER'S DIARRHOEA

### Definition and classification

Diarrhoea and abdominal pain are the most common abdominal symptoms affecting returned travellers, present in up to 85% and 83% of gastrointestinal presentations in this population, respectively [2, 3]. Travellers’ Diarrhoea (TD) is defined as the passage of three or more loose or liquid stools in 24 h, or more frequent passage than normal [4]. This is often accompanied by other abdominal symptoms like nausea, vomiting, faecal urgency, tenesmus, abdominal pain, and bloating.

TD may be classified as acute (lasting <2 weeks), persistent (lasting 2–4 weeks), and chronic (lasting 4 weeks or more) [5]. Given that persistent and chronic TD is often accompanied by other gastrointestinal symptoms, “persistent abdominal symptoms” (PAS) has been suggested as a more appropriate term [6]. Dysentery (bloody diarrhoea), peripheral leucocytosis and female gender were identified as predictors of symptom non‐resolution among travellers with gastrointestinal complaints [3]. While acute TD is frequently only a problem during travel, PAS have been reported in up to 31% of returning travellers who experienced TD during travel [7]. Depending on the exposure time, returning travellers may present with acute TD if they encountered the offending pathogen at the end of their travels.

Functional classification is now preferred wherein TD can be considered mild, if it is tolerable, not distressing, and does not interfere with activities; moderate, if it is distressing and interferes with activities; severe, if it is incapacitating and entirely prevents activity, or if there is any dysentery [8]. Normally, TD is self‐limiting and lasts around 4 days, but can disrupt itineraries in 12%–46% of cases [9, 10]. In severe cases, though, the symptoms are incapacitating, but usually last <24 h [11].

### Epidemiology and risk factors

Although the incidence of TD has decreased in recent years, particularly in regions with growing economies [11], it remains a significant problem for international travellers. The reported incidence varies from 5.4% to 85% [12, 13] of travellers. It is intriguing to consider how COVID‐19 public health measures such as greater use of hand sanitisers may influence the incidence of TD, but an epidemiologic surveillance study from Spain did report a decrease in food and waterborne infections during year 1 of the pandemic [14]. It is difficult to compare data between studies due to varying durations of exposure, population sizes, travel itineraries and attendance at pre‐travel clinics in the relevant studies. Numerous travel, environmental and host factors are known to affect the risk of acquiring TD (Table 1).

**TABLE 1:** Evaluation of travel history in a returning traveller with abdominal symptoms

History component	
Symptoms	Diarrhoea; number of stools per day, functional impairment
	Other abdominal; pain, nausea, vomiting, bloating, urgency, tenesmus
	Musculoskeletal; myalgia, arthralgia
	Systemic; fever, fatigue, weight loss, rashes
Before travel	Pre‐travel health advice received
	Pre‐travel vaccination received, especially for Cholera, Hep A, Typhoid
During travel	Length of travel
	Locations visited
	Chemoprophylaxis for travellers’ diarrhoea or other diseases, e.g., malaria
	Accommodation type and activities esp. any backpacking and freshwater exposure
	Exposure to high‐risk food; street food, tap water, ice cubes, dairy products, shellfish, raw salad, creamy dressings
	Treatment during travel (self‐treatment or otherwise)
	Sick contacts
	Travel sexual history
	History of insect bites

Adapted from [30].

The risk of TD is highest in low‐income countries, where it is the most common illness in travellers [15]. When examining regional risks of TD, countries have been categorised according to the United Nations Sustainable Development Goals regions [16, 17]. Travel to Sub‐Saharan Africa carries a high risk of TD, with rates ranging from 22% to 87% [18, 19]. Rates of TD among travellers to Northern Africa and Western Asia vary from 13% to 41% [18, 20], while the percentage of travellers who acquire TD in Eastern Asia and South‐Eastern Asia range from 15% to 31% [18, 20] and from 16% to 79% [7, 21], respectively. In Central and Southern Asia, the incidences of TD among travellers range from 26% to 52% [18, 20] and from 10% to 82% [7, 22], respectively. Rates of TD in Central America and the Caribbean range between 7% and 55% [23, 24], while in South America they vary from 15% to 44% [20, 23]. Travel to Europe and North America generally carries the lowest risk of acquiring TD, with rates of 3%–13% [25, 26], with the exception of Eastern Europe, in which the risk of TD is comparable to the Caribbean [27]. It is reasonable to assume that the risk of developing TD varies within countries and this may be especially true for larger countries such as Brazil and China, where a gradient of domestic travel health risk must be present but is rarely discussed in literature.

The rates of TD are known to vary at different times of year and are lower in winter months [28]. A study of American students in Mexico found that the risk of acquiring TD increases with higher temperatures and greater quantities of rainfall [24]. Expensive accommodation is not protective, with high rates of TD being found even in some five‐star hotels, in particular following events serving buffet‐style food, which can be exposed to warm environmental conditions [29]. A recent prospective study of adult travellers to Thailand found that eating food from street vendors is associated with a significantly increased risk of acquiring TD [30]. Backpackers and young people tend to have a higher risk of acquiring TD, which may relate to more adventurous behaviour and culinary risk‐taking [11], but also to the propensity of younger people to eat more, which leads to a greater pathogenic load from contaminated food [18].

While pre‐travel vaccination against cholera offers minimal, short‐lived protection against enterotoxigenic *Escherichia coli* in travellers, travellers should be strongly encouraged to attend a comprehensive pre‐travel health consultation for its wider health benefits. Paradoxically, a study found that, not only was their poor adherence with traditional food and water precautions but that TD rates might actually be higher amongst travellers who receive pre‐travel health advice [28]. Nevertheless, while the efficacy of these avoidance strategies for preventing TD is dubious, a history of exposure to high‐risk food and drink is still potentially useful in returning travellers with gastrointestinal problems [31].

## AETIOLOGY OF GASTROINTESTINAL SYMPTOMS IN RETURNING TRAVELLERS

The causes of TD and other gastrointestinal symptoms in returning travellers can be categorised into four groups; infectious causes, presumed infectious causes, post‐infectious sequelae, and unmasked non‐infectious [32].

### Infectious

TD and PAS are generally infectious in origin, although a pathogen is not identified in up to 50% of cases of TD, despite adequate investigations [11]. Infectious causes include bacteria, viruses and parasites, with bacteria being the most common and causing TD in up to 80% of cases [33]. Bacterial pathogens are usually associated with TD that resolves within a week, whereas parasites are more likely to be associated with chronic diarrhoea and PAS [2]. Certain bacteria are also associated with chronic symptoms, including *Campylobacter*, *Salmonella*, and *Shigella* [15].

In a retrospective study of returning travellers with gastrointestinal issues [3], factors associated with a bacterial aetiology included age <37 years on presentation, male gender, C‐reactive protein level >5 IU/dl and white blood cells on stool microscopy, while factors associated with a parasitic aetiology included an absence of bloody diarrhoea and a history of travel to South Asia. A case‐case analysis using prospective data collected from adults presenting with TD in Peru [34], found that while subjective fever was present in 42% of cases, it was most frequently observed with *Campylobacter* (75%), *Shigella* (71%), and enteroaggregative *E. coli* (67%). Vomiting occurred in 34% of cases overall but was most common in patients with enteroaggregative *E. coli* (56%) and norovirus (50%); 10% of patients experienced bloody diarrhoea, most frequently with *Shigella* (36%), *Giardia* (22%) and *Campylobacter* (20%). Table 2 summarises the clinical features of different enteropathogens implicated in TD.

**TABLE 2:** Clinical features of enteropathogens causing travellers’ diarrhoea

Organism	Incubation period	Symptom duration (untreated)	Symptoms
**Bacteria**			
*Enterotoxigenic Escherichia coli*	1–3 days	3–7 days	Abdominal pain, watery diarrhoea
*Enteroaggregative Escherichia coli*	8–52 h	2–4 days	Low‐grade fever, nausea, vomiting, watery diarrhoea often with mucous, abdominal pain, tenesmus, borborygmi, anorexia
*Enteropathogenic Escherichia coli*	4 h	14–120 days	Low‐grade fever, nausea, vomiting, watery diarrhoea
*Campylobacter jejuni*	2–5 days	2–10 days	Fever, abdominal pain, vomiting, diarrhoea with blood or mucous, faecal urgency, tenesmus
*Salmonella* spp.	1–3 days	4–7 days	Fever, abdominal pain, diarrhoea, tenesmus, constipation, fatigue, headache, anorexia, rash, myalgia
*Shigella* spp.	24–48 h	4–7 days	Fever, abdominal pain, vomiting, diarrhoea with blood or mucous, faecal urgency, tenesmus
*Vibrio cholerae*	24–72 h	3–7 days	Life‐threatening dehydration caused by watery diarrhoea and vomiting
*Vibrio parahaemolyticus*	2–48 h	2–5 days	Watery diarrhoea, abdominal pain, nausea, vomiting
*Yersinia enterocolitica*	24–48 h	1–3 weeks	Fever, abdominal pain, nausea, vomiting, diarrhoea with blood
*Clostridium difficile*	5 days–5 months	4 days–months	Fever, abdominal pain, diarrhoea, vomiting
**Viruses**			
*Norovirus*	12–48 h	12–60 h	Fever, watery diarrhoea, abdominal pain, nausea projectile vomiting, myalgia
*Rotavirus*	24–72 h	4–10 days	Fever, nausea, vomiting, watery diarrhoea
**Protozoa**			
*Giardia intestinalis*	9–15 days	Days–months	Fever, abdominal pain, explosive watery diarrhoea, nausea, flatulence, abdominal bloating, anorexia, weight loss chills
*Entamoeba histolytica*	8–30 days	Weeks–months	Fever, diarrhoea with mucus and blood, abdominal pain; chronic type of amoebiasis (amoebic liver abscess)
*Cryptosporidium parvum*	3–12 days	2–14 days	Fever, watery diarrhoea, abdominal pain, nausea, vomiting, weight loss
*Blastocystis hominis*	9–15 days	Days–months	Diarrhoea, abdominal pain, nausea, vomiting, anorexia, fatigue, flatulence
*Cyclospora cayetanensis*	7–10 days	Days–months	Abdominal pain, watery diarrhoea, anorexia, weight loss, bloating, fatigue, flu‐like symptoms
*Cystoisospora belli*	1 week	2–3 weeks	Low‐grade fever, frequent watery diarrhoea, abdominal pain, weight loss
*Dientamoeba fragilis*	1–2 weeks	Acute disease 1–2 weeks; chronic disease 1–2 months	Diarrhoea with mucous, abdominal pain, nausea, vomiting, may be asymptomatic
**Helminths**			
*Schistosoma mansoni*	Weeks–months	2–10 weeks	Fever, bloody diarrhoea, abdominal pain, malaise, myalgia, headache, fatigue
*Strongyloides*	Days–weeks	Weeks–years	Diarrhoea, abdominal pain, nausea, rash, anorexia, constipation

Abbreviation: h: hours.

Adapted from [50, 51].

### Bacteria

Traditionally, enterotoxigenic *E. coli* has been considered the most common cause of TD globally, except in Southeast Asia, where *Campylobacter* has been regarded as the primary culprit [35]. Enterotoxigenic *E. coli* is characterised by the ability to secrete heat‐labile or heat‐stable enterotoxins. Enterotoxigenic *E. coli* is commonly identified in the stools of patients with TD visiting Sub‐Saharan Africa, Northern Africa and Western Asia, Latin America and the Caribbean, being found in up to 11%–42% of cases overall [33, 34].

In recent years, enteroaggregative *E. coli* has become more frequently identified in the stools of patients with TD, with some studies finding higher rates of enteroaggregative *E. coli* than enterotoxigenic *E. coli* in patients from Africa [36] and Latin America [37]. Enteroaggregative *E. coli* has been found in 7%–54% [38, 39] of stool samples of patients with TD, while enteropathogenic *E. coli* has been identified in 8%–44% [38, 39]. Nevertheless, there is ambiguity about the pathogenicity of enteroaggregative *E. coli* and enteropathogenic *E. coli*, as they have a high prevalence in asymptomatic travellers [40]. An analysis of stool samples following travel to West Africa identified that co‐infections occur in up to 79% of cases, with enteroaggregative *E. coli* and enteropathogenic *E. coli* being the most common co‐infections, making it hard to be confident about their pathogenic significance [19]. Recent studies have identified certain genetic and virulence factors in enteroaggregative *E. coli* which may account for heterogeneity in clinical presentation [41, 42], although such evidence is lacking for enteropathogenic *E. coli*.

*Campylobacter* has been found in 3.3%–23.6% of stool samples in patients with TD, with the highest rates reported in Southeast Asia [36, 37]. *Salmonella* and, though generally less common, *Shigella*, overall have been found in 6%–27% [36, 39] and 6%–36% [36, 43] of stool samples, respectively, from patients with TD. *Clostridioides difficile* infection (CDI), though rare, has been implicated in TD in 1.2%–1.4% of cases [44, 45]. One review of published cases of travel‐related CDI found that in 75% of cases for which clinical information was available, patients had taken antibiotics prior to developing diarrhoea, with fluoroquinolones most commonly implicated [46].

### Viruses

Viral pathogens associated with TD include norovirus and rotavirus. Norovirus in particular seems to have increased its frequency relative to other aetiologic agents in recent years and in some studies has been the most commonly identified pathogen [34, 38]. There is also some uncertainty regarding the pathogenic significance of norovirus, given the identification of high amounts of viral shedding in asymptomatic paediatric patients [47]. In another study of TD cases in Thailand, norovirus was found in a significantly greater number of patients with TD than in controls, supporting its role in disease [48]. A prospective study of pre‐ and post‐travel stool samples in travellers analysed by quantitative polymerase chain reaction (PCR) found a low rate of rotavirus infection (0.7%) [49]. An earlier study of French paediatric travellers detected rotaviruses in 15%; the higher rate is presumably the result of low rotavirus vaccine coverage in their country [39]. SARS‐CoV‐2 causes gastrointestinal symptoms of diarrhoea and abdominal pain in some patients, putatively owing to the presence of angiotensin‐converting enzyme‐2 receptors in the gut, although this appears to occur less frequently with more recent viral variants. It should be entertained in the differential diagnosis of symptomatic returned travellers.

### Parasites

Parasites are more likely to be associated with chronic diarrhoea and PAS [2]; one of the most common parasites implicated in TD is *Giardia*, which has been identified in 6%–14% [39, 50] of stool samples. An earlier study examined the causes of persistent TD and found that *Giardia lamblia* was the most common organism identified, found in 16.4 % of cases, followed by *Blastocystis hominis*, which was found in 9.6% of cases; *Cyclospora* (3.5%) and *Entamoeba histolytica* (0.9%) were identified in 3.5% and 0.9% of cases, respectively [51]. In a more recent retrospective study of patients presenting with a history of travel and abdominal symptoms lasting for longer than 2 weeks, 55% of cases had an organism identified in stool samples and, of these, 85% were parasitic [5].

### Post‐infectious

#### Post‐infectious irritable bowel syndrome

TD can significantly increase the risk of patients developing functional gastrointestinal disorders, especially post‐infectious irritable bowel syndrome [11, 17]. Irritable bowel syndrome can be defined according to Rome IV criteria [54]. Two meta‐analyses have demonstrated a six to seven times greater risk of developing irritable bowel syndrome following an episode of gastroenteritis [55, 56], and post‐infectious irritable bowel syndrome occurs in up to 17% of patients who experience TD [57, 58]. Factors that have been identified as increasing the risk of developing post‐infectious irritable bowel syndrome after TD include female gender, younger age, infection with heat‐labile‐toxin‐producing enterotoxigenic *E. coli*, longer length of travel, and greater severity of symptoms [6, 59]. In a prospective study examining gastrointestinal symptoms in returning travellers, anxiety and greater severity of symptoms during an episode of TD contributed to an increased risk of developing functional gastrointestinal disorders [60].

#### Presumed infectious

In rare cases, diseases of uncertain aetiology that are presumed to be infectious in nature can cause gastrointestinal issues in returning travellers, including Brainerd's diarrhoea and tropical sprue [32]. The risk factors for Brainerd's diarrhoea include consumption of contaminated water and unpasteurised milk, with the majority of cases being described in the United States [61]. Tropical sprue is a chronic enteropathy characterised by malabsorption, with diarrhoea, weight loss, steatorrhoea, fatigue and micronutrient deficiencies; it is endemic in tropical regions [62].

#### Unmasked non‐infectious

Coeliac disease is the most common small bowel enteropathy in Western countries and is caused by an immunological reaction to dietary gluten. It presents with a malabsorptive syndrome of diarrhoea, steatorrhoea, weight loss, abdominal pain, bloating, and various extraintestinal complications, and can occasionally be diagnosed in returning travellers with chronic abdominal symptoms [5].

It is suggested that the risk of developing IBD increases after acute infectious gastroenteritis [63], and IBD has been diagnosed in returning travellers with chronic gastrointestinal symptoms [32]. Colorectal cancer has been identified in returning travellers with PAS [5]. Guidelines recommend that patients with a change in bowel habit be sent on the suspected cancer referral pathway if they are aged 60 years or older, or are aged under 60 years and have unexplained rectal bleeding [64]. Other causes of persistent diarrhoea in individuals returning from travel abroad include non‐biological contaminants in food and water, use of recreational drugs, and medication use, including excessive use of laxatives, for example, in children who suffer constipation during a family trip overseas.

## DIAGNOSIS

### Microbiological diagnosis

Although the aetiology of TD is usually infectious, it is not always necessary to obtain a definitive microbiological diagnosis, given that the condition is typically self‐limiting and often responds well to empiric antimicrobial therapy [11]. Guidelines recommend, however, that microbiological diagnosis is sought in the context of PAS, or severe symptoms, including the presence of mucus or blood in the stool (dysentery) [8].

Bacterial aetiologies have traditionally been identified using culture‐based methods applied to stool samples, which are limited by their low sensitivity and the longer time before results are available [65]. Parasites have historically been detected following microscopic examination of either fresh stool or stool that has been stained, looking for oocytes, cysts and parasites; this technique is time‐consuming and lacks sensitivity. Antigen tests have been developed for a limited number of parasites, including *Giardia intestinalis*, *Cryptosporidium* spp. and *Entamoeba histolytica*, as well as viruses such as adenovirus and rotavirus, and the bacterial toxin produced by *C. difficile* [8]. Performing investigations on multiple stool samples increases the diagnostic yield since microorganisms can be shed intermittently in faeces [2, 5].

In the past decade, molecular‐based clinical assays have been developed, including multiplex PCR tests, which can test for multiple bacterial, viral and parasitic organisms simultaneously. The xTAG® can detect 15 enteropathogens, with evidence of particularly high sensitivity for *Shigella, Giardia* and enterotoxigenic *E. coli*, however, it does not test for enteroaggregative *E. coli* or *Cyclospora cayetanensis* [44]. The FilmArray™, by comparison, tests for 22 enteropathogens including enteroaggregative *E. coli* and *Cyclospora cayetanensis*, and results can be yielded in 1 h, whereas results take up to 5 h for the xTAG®[66].

It has been suggested that the increasing number of TD cases associated with norovirus, enteroaggregative *E. coli* and enteropathogenic *E. coli* globally might be linked to improved detection with highly sensitive molecular techniques [17]. Nevertheless, isolation of a microorganism from a non‐sterile part of the body does not necessarily imply pathogenic significance [11], and a challenge that arises, therefore, from the use of highly sensitive molecular techniques is to be able to determine whether detected organisms are the cause of a patient's symptoms. Data from case‐control studies suggest that potentially pathogenic organisms are detectable in close to half of asymptomatic controls [43, 48, 50]. Quantitative PCR studies have been suggested to differentiate colonisation from infection and thereby better understand the pathogenic significance of different microorganisms in the context of TD and PAS [67].

The impact of molecular diagnostics in terms of clinical outcome and cost‐effectiveness is poorly understood. In a retrospective chart review of outpatients with vomiting and diarrhoea, use of multiplex PCR only changed clinical management in 5.2% of cases [68]. Guidelines are unable to provide evidence‐based advice regarding the use of molecular methods; however, it is generally accepted that these techniques are preferred where rapid results are required, or where non‐molecular tests have failed [8]. Traditional culture‐based methods remain essential for determining antibiotic susceptibility profiles [17].

### Non‐microbiological diagnosis

If symptoms are severe or persistent and no microbiological diagnosis can be made, a comprehensive workup for non‐infectious causes should be performed. While post‐infectious irritable bowel syndrome is a diagnosis of exclusion, coeliac disease is diagnosed based on serologic testing and a confirmatory duodenal biopsy [69]. Inflammatory bowel disease is diagnosed based on endoscopic mucosal biopsy. Endoscopy can also identify any gross pathology such as tumour and abdominal CT scans may be helpful in certain cases to further assist with reaching a diagnosis [32].

## MANAGEMENT

### Travellers’ diarrhoea

Acute TD as well as persistent or chronic TD can present in returning travellers depending on the time of exposure to the aetiologic agent. Management of TD varies according to the severity of the disease but generally aims to avoid dehydration, limit symptoms and mitigate disruption to itineraries [11]. Although targeted antimicrobial therapy can be given if a causative pathogen is identified, searching for a microbiological diagnosis is only recommended in severe cases or cases with chronic symptoms [8]. In acute TD, due to the preponderance of bacterial cases, antibiotics are the first‐line empiric treatment, although they are not required in all cases [8].

### Mild TD

For cases of mild TD, antibiotic therapy is not recommended; patients are advised to use supportive measures like oral rehydration and anti‐diarrhoeal agents [8]. Antibiotics may be beneficial in the treatment of TD, shortening the symptom duration from 50 to 93 h to <36 h [70] and, when used in conjunction with loperamide, to <12 h [71]. There is concern about a correlation between antibiotic use in travellers and the spread of antimicrobial resistance, in particular an increased risk of acquiring extended‐spectrum beta‐lactamase‐producing *Enterobacteriaceae* (ESBL‐PE) [72]. Additionally, the use of empiric antibiotics in travellers is associated with a risk of CDI [46]. For these reasons a judicious approach to the use of antibiotics for patients with TD, especially mild TD, is important. Table 3 summarises regional rates of AMR among enteropathogens globally.

**TABLE 3:** Regional rates of antibiotic resistance among enteropathogens (references in parentheses)

Antibiotic	Global, %	North Africa and Western Asia, %	Sub‐Saharan Africa, %	East and Southeast Asia, %	Southern and Central Asia, %	Latin America and the Caribbean, %	Europe and North America, %
Ampicillin	35.5 (88)	28.1–75.7 (89, 90)	17.0–50.0 (90)	‐	0.0–49.4 (82, 88, 90)	0.0–60.0 (82, 88, 90)	‐
Azithromycin	5.0 (88)	‐	0.0–25.0 (88, 91)	28.6–33.3 (91)	0.0–24.5 (78, 82, 88)	0.0–50.0 (80, 82, 88, 91)	‐
Cefotaxime	2.6–96.0 (88, 92)	‐	0.0–6.3 (88, 91)	33.3–42.9 (91)	3.3 (88)	0.0 (88, 91)	‐
Ceftriaxone	‐	‐	‐	‐	0.0–6.2 (82)	0.0–20.0 (82)	‐
Ciprofloxacin	27.6–61.0 (79, 88, 92)	66.7 (79)	2.7–52.0 (79, 88)	41.7–68.0 (79, 91)	0.0–76.1 (78, 79, 88)	0.0–89.8 (79, 80, 82, 91)	56.1 (79)
Doxycycline							
Erythromycin							
Levofloxacin	‐	‐	‐	‐	0.0–40.8 (82)	0.0–45.0 (82)	‐
Rifaximin	‐	‐	‐	‐	0.0–29.4 (82)	0.0–15.5 (82)	‐
Trimethoprim/sulfamethoxazole	82.4 (88)	25.0–100.0 (89, 90)	8.0–88.9 (88, 90, 91)	35.7–50.0 (91)	0.0–75.9 (78, 82, 88, 90)	0.0–75.0 (82, 88, 90, 91)	‐

Adapted from [17].

There is more evidence supporting the use of loperamide to treat mild TD [8]. In one study of acute TD among 219 students visiting South America, patients who received loperamide passed fewer unformed stools compared to patients receiving BSS, in the first 48 h after treatment [73]. Some concerns taking anti‐diarrhoeal medication increases patients’ intestinal exposure to pathogens, however, these are largely unsubstantiated, with a randomised trial comparing the use of rifaximin, loperamide and a rifaximin‐loperamide combination therapy finding that all treatment options had a low risk of adverse events [74]. Using antibiotic and loperamide together has been associated with increased acquisition of ESBL‐PE [75]. Although adverse events are rare, cessation of loperamide therapy should be considered in the presence of worsening, severe or invasive symptoms like dysentery, severe abdominal pain, and high fever [8].

### Moderate TD

Current guidelines recommend considering the use of antibiotics for moderate TD and loperamide may also be used, either alone or in combination with antibiotics [8]. For many years, trimethoprim‐sulfamethoxazole was the empiric antibiotic of choice [76]; however, increasing resistance diminished its usefulness [77] and recommended antibiotics now include azithromycin and rifaximin [8]. Previously, fluoroquinolones were the preferred antibiotic in most regions, except Southeast Asia, where resistant *Campylobacter* species predominated [11, 78]. There is emerging evidence of fluoroquinolone resistance in various regions, especially in North Africa and Latin America [79, 80]. There are uncertainties about the risk‐benefit ratio of the use of fluoroquinolones in moderate TD [8], despite randomised controlled trial evidence of their efficacy in this setting [70, 81].

Azithromycin has been shown to be as efficacious as fluoroquinolones for the treatment of moderate TD [81] and, although there have been reports of increasing concentrations being required to inhibit growth of enteroaggregative *E. coli* and enterotoxigenic *E. coli* [82], there is less of a problem of antimicrobial resistance [17]. Despite this, nausea and vomiting are more frequently encountered with azithromycin [83] and it can also lead to sustained ventricular tachycardia in patients with a prolonged Q‐T_c_ interval, although a meta‐analysis has found that there is no increased risk of cardiovascular events or mortality [84].

Rifaximin is an oral antibiotic which has been found to be more effective than placebo in the treatment of moderate TD caused by non‐invasive pathogens, and is as efficacious as fluoroquinolones for this purpose [81]. Rifaximin has a favourable safety profile, with far less evidence of increasing antimicrobial resistance compared to azithromycin and fluoroquinolones [85]. There is a suggestion that rifaximin, which improves symptoms of irritable bowel syndrome, could have beneficial effects on bowel microbiomes [86]. Rifaximin should be avoided in cases of TD caused by a known invasive organism (e.g., *Campylobacter*, *Shigella*) or with invasive features like dysentery, severe abdominal pain or high fever [8].

### Severe TD

It is recommended to treat cases of severe TD with antibiotics; azithromycin is preferred, though fluoroquinolones and rifaximin can be considered in cases of severe non‐dysenteric TD [8]. Antibiotics are proven to be effective in treating moderate‐severe cases of TD [17, 87], however, many of the studies include patients with TD across a spectrum of severity and may exclude more severe cases with dysentery or requiring hospitalisation.

### Novel therapies

Rifamycin SV‐MMX® (RIF‐MMX) has emerged as a promising new therapy for treating TD [93]. RIF‐MMX is an antibiotic formulation with the active ingredient of rifamycin SV; rifamycin SV is an oral poorly absorbable broad‐spectrum antibiotic, which is closely related to rifaximin. The active ingredient in RIF‐MMX is only released an hour after reaching pH > 7, which targets delivery to the distal small bowel and colon [94]. It is believed that more than 90% of cases of TD are non‐dysenteric and caused by non‐invasive enteropathogens, and so can be safely treated with poorly absorbed antibiotics like rifaximin or RIF‐MMX [95]. In vitro studies have demonstrated the efficacy of rifamycin‐SV against organisms commonly implicated in TD and against *C. difficile* [96].

There is promising evidence to support the use of novel therapies, including phages and bacteriocins in treating a variety of infections, including TD [97]. The *Myoviridae* phage PDX effectively killed enteroaggregative *E. coli* in both in vivo mouse models and in vitro human faeces [98]. The antimicrobial peptide microcin J25 was found to reduce enterotoxigenic *E. coli*‐induced weight loss, diarrhoea and intestinal inflammation in a mouse model [99].

## NON‐DIARRHOEAL GASTROINTESTINAL PROBLEMS

Abdominal pain is the second most common abdominal symptom experienced by returning travellers, observed in up to 83% of presentations [2]. Abdominal emergencies should be excluded in any patient presenting with acute abdominal pain. A detailed review of the acute abdomen is available elsewhere [100]. Infectious aetiologies predominate in returning travellers presenting with persistent abdominal pain and other forms of PAS, with parasites most often responsible [5]. A retrospective study of 102 returning travellers with PAS and a negative stool test found that empirical treatment with the anti‐parasitic agents tinidazole and albendazole resulted in symptom improvement in 69% of cases, with full symptom resolution in 37% [2]. While this study was limited by the absence of a control group, most of the patients had already undergone failed treatment with antibiotics, antacids and irritable bowel syndrome therapies. This provides justification for the empiric use of anti‐parasitic agents in returning travellers with PAS, for whom no infectious cause can be found before intensive investigation for non‐infectious causes begins.

**FIGURE 1:**
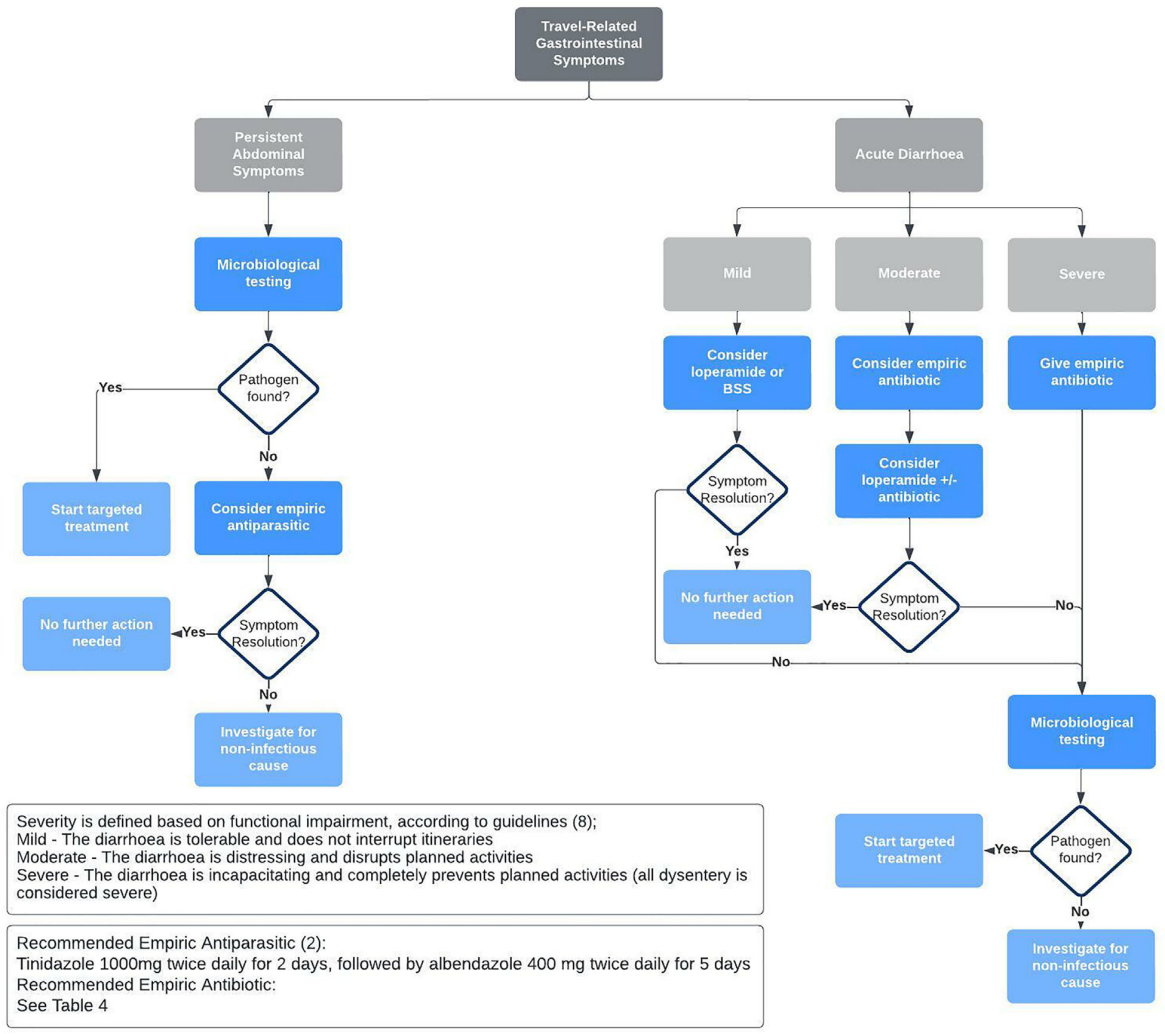
An approach to returning travellers with abdominal symptoms, with a focus on bacterial TD.

Tinidazole is better tolerated and required for a shorter duration than metronidazole [101] and may be more effective in the treatment of PAS [5]. Including albendazole in the treatment is useful as it is effective against both helminths and protozoa, and the combination of tinidazole and albendazole may be effective against resistant types of *Giardia* [102]. Figure 1 provides a summary of the approach to returning travellers with abdominal symptoms. A summary of empiric antibiotic therapy for patients with acute TD is given in Table 4.

**TABLE 4:** Recommended empiric antibiotics for acute travellers’ diarrhoea

Antibiotic	Dose	Duration	Comment
Azithromycin	1000 mg PO or	Single or 1‐day divided	First line antibiotic in Southeast Asia and cases of severe diarrhoea
	500 mg PO	3 days	
Ciprofloxacin	750 mg PO or	Single dose	‐
	500 mg PO	3 days	‐
Levofloxacin	500 mg PO	Single dose or 3 days	‐
Ofloxacin	400 mg PO	Single dose or 3 days	‐
Rifaximin	200 mg PO TDS	3 days	Contraindicated in cases of invasive diarrhoea

Abbreviations: PO: per os (by mouth); TDS: ter die sumendum (three times a day).

Adapted from [8].

## GUT DYSBIOSIS

Far from being simple intestinal passengers, there is growing evidence that the microorganisms of the gut microbiome play active roles in health and disease; they contribute towards digestion, including the processing of vitamins and xenobiotics, protect the gut from pathogen invasion, and interact with the body's immune system [103, 104]. Disturbances in the gut microbiome have been associated with diseases like inflammatory bowel disease and diabetes [105].

A retrospective bacterial 16S rDNA sequencing study used stool samples from US travellers to South America [106]. It found that TD was associated with a dysbiotic gut microbiome profile characterised by a high *Firmicutes‐Bacteroidetes* ratio. While the microbiota of healthy travellers was similar to those with TD, when compared with healthy samples from the Human Microbiome Project, there was a significantly higher *Firmicutes‐Bacteroidetes* ratio. This seemed to suggest that travelling could be associated with gut dysbiosis independent of TD [106]. Another study [107] found contrasting results to the previous study [106], however, with travel from the United States to Southeast Asia being associated with a nearly two‐fold increase in the *Bacteroidetes‐Firmicutes* ratio that reversed upon return [107].

A prospective observational study of Swedish travellers found that, while international travel did not change the overall diversity of faecal microbiota, it was associated with an increased *Enterobacteriaceae‐Christensenellaceae* ratio [108]. This is significant as *Enterobacteriaceae* have been associated with intestinal inflammation and diseases like coeliac disease [109], while *Christensenellaceae* have been linked with good health and may even be protective against obesity [110].

Variations in the gut microbiota in travellers may affect the risk of acquiring TD. The risk of acquiring TD was greater in the presence of a higher relative abundance of *Prevotella copri* before travel. Diarrhoea during travel was associated with a less diverse microbiome on return compared to travellers without diarrhoea [111]. They also found that a greater abundance of *Bifidobacterium* and *Clostridiales* strains was associated with a smaller chance of persistent carriage of travel acquired multidrug‐resistant *Enterobacteriaceae* at 1 month [111]. Lower pre‐travel microbiome diversity was associated with a greater likelihood of persistent carriage of travel‐acquired multidrug‐resistant *Enterobacteriaceae* at 1 month [111].

Further research is needed to truly understand how travel to different areas can alter an individual's gut microbiome, and how these changes might affect chances of developing TD and acquiring multidrug‐resistant microorganisms. Exciting potential applications of this knowledge could include the use of probiotics to optimise the microbiome before travel but there is insufficient evidence currently to recommend this practice [8].

## SPECIFIC GROUPS OF TRAVELLERS

### Visiting friends and relatives (VFRs)

The frequency of TD seems to be lower in this population than in other groups of travellers, with one study of 351 VFRs finding a rate of TD of 4.8% [112]. This is possibly due to residual immunity from previous time spent in high‐risk areas. Nevertheless, within this group of travellers, VFR children have been identified as being at high risk for developing TD, which may relate to a deficiency in pre‐travel health advice and a lack of precautions during travel [113].

### Military personnel

TD represents a significant source of morbidity in this population, with reported rates varying from 10% to 42% [114, 115]. Specific risks include eating outside the military base and having a colleague with diarrhoea [116, 117]. TD among military personnel is a concern due to its capacity to interfere with operational capacity [117].

### Immunosuppressed patients

Guidelines recommend that travellers who are immunosuppressed, including those taking immunosuppressive medication for IBD, bring on‐demand antibiotics due to a higher likelihood of infection [118], and antimicrobial prophylaxis for TD can also be considered in these patients [8]. There is little evidence that immunocompromised patients experience TD more frequently than other travellers, although the symptoms seem to be more severe [119]. Nevertheless, patients with IBD have a higher rate of morbidity while travelling and can experience flare‐ups [120].

### Healthcare students

While on international electives, healthcare students will frequently engage in cultural side trips and may do adventurous activities as well [121]. In a GeoSentinel study of illnesses among 432 returning United States medical students, gastrointestinal illnesses were the most common, with acute diarrhoea predominating [122]. Reported rates of diarrhoea among medical students participating in international electives vary from 48% to 69% [123, 124].

### Business travellers

Before the COVID‐19 pandemic, business travel accounted for approximately 14% of international travel volumes [125]. International business trips fluctuate greatly in terms of location visited and length of stay. A GeoSentinel study of 12,203 unwell business travellers found that acute diarrhoea was the most common syndrome, occurring in 24% of cases, while chronic diarrhoea and other gastrointestinal complaints each occurred in 8% of cases [126].

## STRENGTHS AND LIMITATIONS OF REVIEW

Our search strategy prioritised articles published in the last 5 years, but seminal material published earlier was also included where a more recent source was unavailable. Our literature search was restricted to articles published in the English language, and potentially relevant studies published in other languages may have been excluded.

## CONCLUSION

Gastrointestinal issues are among the most common health problems experienced by returning travellers. Diarrhoeagenic *E. coli* species remain the most common causes of TD, with increasing frequencies of cases caused by enteroaggregative *E. coli* and enteropathogenic *E. coli*. Molecular diagnostic techniques enable rapid and highly sensitive testing for a range of enteropathogens, though more research is required to confirm the pathogenic significance of identified organisms. While travel clearly influences the microbiome, further research is needed to accurately determine the changes that occur and clarify the underlying mechanisms.

## AUTHOR CONTRIBUTIONS

Gerard T. Flaherty was responsible for study conception, study design, literature screening, and editing of subsequent drafts of the manuscript for significant intellectual content. Dominic Butler was responsible for the literature search, preparation of the first draft of the manuscript and editing of subsequent drafts. Ramona McLoughlin was responsible for reviewing and editing of manuscript drafts. All authors read and approved the final version of the manuscript.

## CONFLICT OF INTEREST

None declared.

## ETHICS APPROVAL

Not required.

## Data Availability

Data sharing is not applicable to this article as no new data were created or analysed in this study.
